# Generation mechanism of hydroxyl radical species and its lifetime prediction during the plasma-initiated ultraviolet (UV) photolysis

**DOI:** 10.1038/srep09332

**Published:** 2015-03-20

**Authors:** Pankaj Attri, Yong Hee Kim, Dae Hoon Park, Ji Hoon Park, Young J. Hong, Han Sup Uhm, Kyoung-Nam Kim, Alexander Fridman, Eun Ha Choi

**Affiliations:** 1 Plasma Bioscience Research Center, Kwangwoon University, Seoul, Korea; 2 Yonsei University, Seoul, Korea; 3 Drexel Plasma Institute, Drexel University, PA, USA

## Abstract

Through this work, we have elucidated the mechanism of hydroxyl radicals (OH^•^) generation and its life time measurements in biosolution. We observed that plasma-initiated ultraviolet (UV) photolysis were responsible for the continues generation of OH^•^ species, that resulted in OH^•^ to be major reactive species (RS) in the solution. The density and lifetime of OH^•^ species acted inversely proportional to each other with increasing depth inside the solution. The cause of increased lifetime of OH^•^ inside the solution is predicted using theoretical and semiempirical calculations. Further, to predict the mechanism of conversion of hydroxide ion (OH^−^) to OH^•^ or H_2_O_2_ (hydrogen peroxide) and electron, we determined the current inside the solution of different pH. Additionally, we have investigated the critical criterion for OH^•^ interaction on cancer cell inducing apoptosis under effective OH^•^ exposure time. These studies are innovative in the field of plasma chemistry and medicine.

Recently, nonthermal atmospheric pressure plasma (NTP) has been investigated as a novel tool in the field of plasma bioscience and medicine. Especially, a lot of various reactive oxygen species (ROS) could be generated inside the biosolution during NTP jet bombardment onto the solution. NTP jet has recently been used and known to be very promising in sterilization, chronic wounds, tumor, and diabetic treatment^1^,^2^,^3^,^4^,^5^. These functions of plasma may be possibly resulted from the behaviors of various reactive chemical species generated inside the biosolution during NTP bombardment onto the solution. It is well known that chemically ROS^6^,^7^,^8^,^9^ and reactive nitrogen species (RNS)^10^,^11^,^12^ play an important role in many different body systems, which may explain the biomedical effects by NTP treatment. Moreover, interactions of the NTP with biosolutions have received increasing attentions for their biomedical applications, since the biological cells inside the biosolution have been found to be either killed or stimulated by the various amount of ROS or RNS produced according to the employing plasma gases, discharge power, plasma exposure times, and other experimental conditions. These effect of ROS and RNS on the biological cells being on the solutions and being in the solutions^6^,^7^,^8^,^9^,^10^,^11^,^12^ are not also studied well yet. ROS and RNS species such as hydroxyl radical (OH^•^)^6^,^7^,^8^,^9^, hydrogen peroxide (H_2_O_2_)^13^, superoxide anion (O_2_*^−^)^14^,^15^, as well as nitric oxide (NO)^5^,^10^,^11^,^12^,^16^ species could be generated on the biosolution surface by NTP jet bombardment.

Other research groups previously studied the direct or indirect photoionization by using a high energy photon greater than 8 eV^17^,^18^,^19^, and middle energy photoionization by using a photon whose energy is ~6 eV^20^ without NTP. However, we have here investigated the plasma-initiated UV photolysis with low energy ~4 eV, especially for the clarification of the OH^•^ generation mechanism as well as H_2_O_2_ inside the biosolutions. The OH^•^ density inside the biosolutions has been investigated using ultraviolet absorption spectroscopy (UVAS) during the Ar NTP jet irradiation. The visual confirmation of OH^•^ and H_2_O_2_ generation in biosolutions has also been studied using chemical assays. Additionally, we have tried to comprehend the generation of electron (e^–^) and OH^•^ from hydroxide ion (OH^−^) by measuring the current in solutions of different pH. Further, we determined that the formation of H_2_O_2_ is more in basic solution as compared the neutral and acidic solution using titanyl ions test.

We have also investigated the influence of OH^•^ densities on the lung cancer cells (H460) inside the biosolutions when the Ar plasma jet has been irradiated on biosolutions. The ion-induced secondary electron emission coefficient and molecular surface energy distribution of the H460 cells have also been investigated by using He^+^ ion beam whose energy is less than 200 eV. Here we have investigated and suggested a threshold criterion, nT, for apoptosis of H460 cells adhered on petri-dish of the phosphate buffered saline (PBS), where n is OH^•^ density and T is effective plasma exposure time on cell, under Ar plasma treatment by 60 s at 2 mm depth in PBS with the OH^•^ density of ~1.9 × 10^16^ cm^−3^ in this experiment.

## Results

### Plasma-initiated photolysis in biosolutions: Simultaneous generation of OH^•^ species inside the DI water during plasma bombardment onto the water

**Figure 1:**
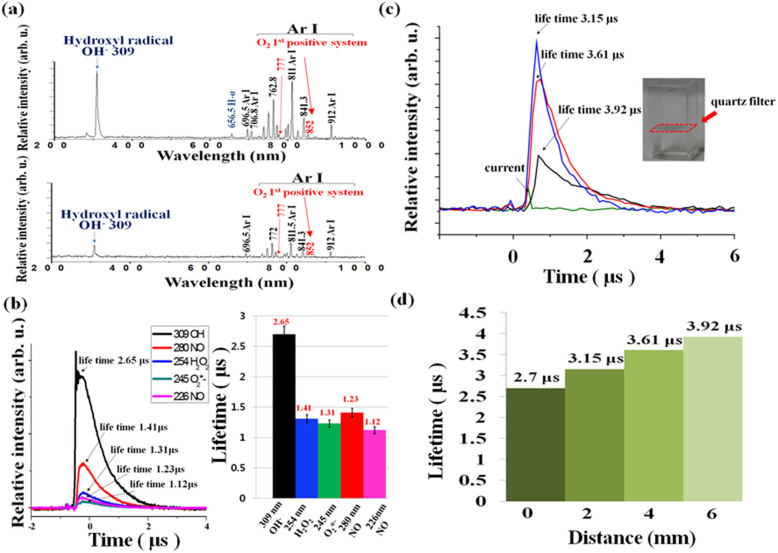
(a) Optical emission spectrum measured by CCD spectrometer with optical fiber at the 2 mm above and below region, respectively, of the DI water under Ar plasma jet bombardment onto the biosolution surface with inclusion of Ar gas in glove box; (b) Lifetimes for various ROS measured at ambient air region of 2 mm above the water surface without Ar glove box by using the monochromator; (c) Temporal behaviors of 309 nm for OH^•^ emission intensity for different depth locations of 2 mm, 4 mm, and 6 mm inside the DI solution with quartz filter located at 1 mm depth position of water during Ar plasma jet bombardment; (d) Lifetimes of OH^•^ vs water depth positions for 2 mm, 4 mm, and 6 mm from the surface.

Especially, optical emission spectroscopy (OES) above and inside DI water for NTP jet bombarding onto the surface could be exploited for investigation of various kinds of induced ROS. Figure 1(a) shows the OES measured by CCD spectrometer at the 2 mm above (upper) and below region (below) respectively, of the DI water, with the inclusion of Ar gas in the glove box under Ar NTP jet bombardment onto the solution. It was apparent that the OH^•^ just above the water surface had strong emission lines at 309 nm, while no lines for N_2_ second positive system (C^3^Π_u_–B^3^Π_g_) were observed in the wavelength range of 320–380 nm. While, the emission lines at 224 nm and 245 nm for NO and O_2_*^-^, have been observed under air environment without Ar glove box, respectively. On moving 2 mm below the DI water surface under Ar gas environment, the emission lines around 309 nm for OH^•^ diminished in comparison to that above the surface^7^. To our surprise, the lines for the NO, O_2_*^-^, and N_2_ second positive system (C^3^Π_u_–B^3^Π_g_) were not observed. Figure 1(a) clearly display the emission lines at 777 nm and 852 nm inside the water (below), these lines were originated from the atomic oxygen (OI 5S0 - 5P) and O_2_ first positive lines, respectively and the same being more diminished for inside than those above the water.

These radicals generated in the ambient air environment can initiate many reactions in biosolution, some of them are following:


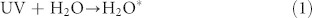

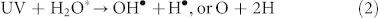

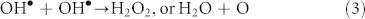

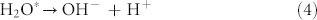

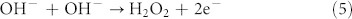

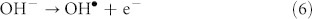

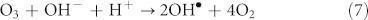


In the ambient air phase, the OH^•^ + OH^•^ → H_2_O_2_ has reaction coefficient of 1.78 × 10^−11^ cm^3^/mole/s, and OH^•^ + H_2_O_2_→ HO_2_ + H_2_O is 1.7 × 10^−12^ cm^3^/s^21^, hence both reactions play important role in plasma chemistry inside water. Figure 1(b) shows the lifetimes for various ROS of OH^•^ (309 nm), NO (280 and 226 nm), H_2_O_2_ (254 nm), and O_2_*^−^ (245 nm), respectively, measured at ambient air region of 2 mm above the water surface without Ar glove box by using the monochromator with focal length 30 cm and photomultiplier tube with fast operational amplifier. It is observed here that these ROS are simultaneously generated with respect to the discharge current (left in Figure 1(b)), and OH radical has shown to be the longest lifetime of ~2.7 μs among these ROS. Also the lifetimes are measured to be ~1.2 μs for NO, 1.4 μs for H_2_O_2_, and ~1.3 μs for O_2_*^−^ (right in Figure 1(b)), respectively. From these observed results of ROS in ambient air of water surface, we see that there are strong plasma-initiated ultraviolet (UV) emissions ranged from ~220 nm (~6 eV) to 309 nm (~4 eV) excited by these ROS. These plasma-initiated UV's will propagate into the DI water from the water surface to excite and dissociate the H_2_O molecules into OH^•^ species and H_2_O_2_, which has been done mainly due to 309 nm with energy of ~4 eV.

Further, we did experiments to examine the continuous generation of OH^•^ at different depth positions of the DI water by Ar plasma jet bombardment onto the solution. The Figure 1(c) shows the OH^•^ emission lines around 309 nm were investigated at the respective depth region of 2 mm, 4 mm, and 6 mm below water. These OH^•^ emission lines have been measured, that are simultaneously generated with respect to the discharge current and reached to their peak after ~100 ns under discharge voltage of 2.8 kV at different depth locations (2 mm, 4 mm, and 6 mm). The attached quartz filter is to screen the plasma electrons, ions, and neutral particles. Hence, only plasma-initiated UV emitted from the excited species of ROS on the water surface could pass the filter for propagation into the water. Figure 1(c) shows that the peak at 309 nm for OH^•^ has been diminished as the depth position was increased from 2 mm to 6 mm. This will be correlated later to the OH^•^ densities according to the depth positions. Figure 1(d) shows OH^•^ lifetime versus the depth positions inside the water. The OH^•^ lifetime has been increased from 2.7 μs on the surface (0 mm) to 3.15 μs, 3.61 μs and 3.92 μs with increasing depth at 2 mm, 4 mm, and 6 mm, respectively. From these Figures 1(c) and (d), the OH^•^ species inside the DI water might be generated simultaneously by plasma-initiated UV photolysis, which is propagating into the DI water from the water surface. Throughout the UV photolysis of H_2_O molecules in the DI water, which has been mainly caused by 309 nm with energy of about 4 eV, H_2_O molecules could be ultimately dissociated into OH^•^ species and H_2_O_2_ by continuous UV exposures to H_2_O and their excited molecules inside the DI water.

To have a clear understanding of increased lifetime, we studied the hydrogen bonding calculation between OH^•^ and H_2_O using semiempirical calculations with the help of Hyperchem 7^22^,^23^ and the interactions are explicitly elucidated as in Figure S1 and Table S1. We have chosen 2 OH^•^ and 40 water molecules for the first calculation that resemble OH^•^ inside depth of water ~6 mm (assumption for the less OH^•^ and more water molecules at depth of ~6 mm, as shown in Figure S1). The estimated hydrogen bond energy was predicted as ~126.603 kcal/mol. In another case, we choose 4 OH^•^ and 40 water molecules to resemble the condition at ~4 mm (assumption: Figure S1) that density of OH^•^ being higher at 4 mm as compared to the 6 mm (as discussed later). Further, we estimated hydrogen bond energy 37.64 kcal/mol from the calculations. This reveals estimated hydrogen bond energy is less at higher density of OH^•^ resulting in shorter lifetime. Hence, these findings support our experimental and theoretical studies for longer lifetime of OH^•^ at 6 mm depth.

### Visualization of OH^•^ and H_2_O_2_ generation inside the DI water induced by plasma-initiated UV photolysis

**Figure 2:**
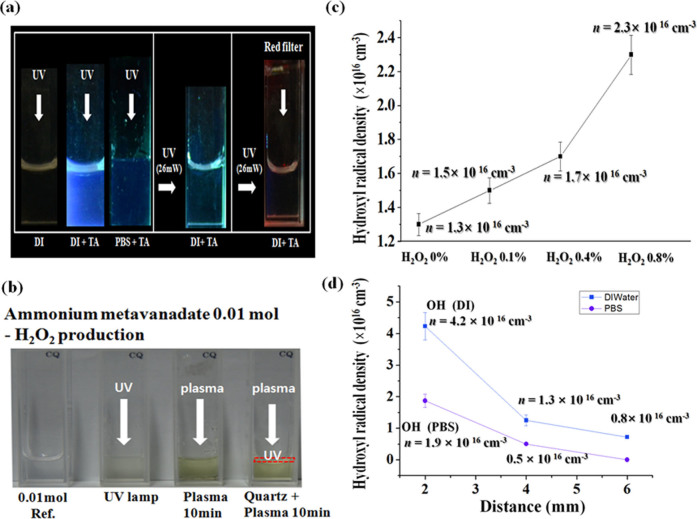
(a) Visual observation for generation of OH^•^ species inside the DI water and PBS when the UV mercury lamp has been irradiated onto their surface; (b) Visual confirmation for generation of H_2_O_2_, inside the DI water either by irradiation of UV or plasma bombardment onto the water surface with quartz filter located just below the DI surface; (c) OH^•^ density vs the external H_2_O_2_ concentrations in DI water at 4 mm depth position, when the Ar plasma jet has been bombarded; (d)Density of OH^•^ vs the depth positions of the DI water and PBS solutions, generated by Ar plasma jet operated in Ar glove box, under the low electrical power of 4.9 W and driving frequency of 35 kHz.

Here we have investigated the generation of OH^•^ species by visual check inside the DI water by irradiation of UV onto the water surface. In this experiment, the UV has been produced by mercury lamp whose wavelength has been centered at 306 nm. We have put the chemical indicator of terephthalic acid (TA)^24^ into the DI to see visually whether OH^•^ has been produced in the biosolutions during UV bombardment^7^. If the OH^•^ has been produced in the biosolutions, then their colors will be changed to blue for visual check. It is noted in this experiment that the spectral ranges in DI water for mercury UV lamp are above wavelengths of 270 nm, however, they are above 295 nm for inclusion of TA in DI water and PBS. Figure 2(a) shows that the OH^•^ species could be generated inside the DI water and PBS when the UV mercury lamp has been irradiated onto their surface since their colors are changed to blue in this experiment. Here we could confirm that the OH^•^ could be generated inside the DI water and PBS by UV bombardment, whose energy is centered at ~4 eV, onto the surface with or without quartz filter (dotted region in Figure 2(a)) placed at 1 mm below the water surface. This UV could pass the filter for propagation into the DI water for excitation and dissociation of water molecules, resulting in OH^•^ generation by continuous exposures to solutions. We have also performed visual observation whether the OH^•^ could be generated inside the water either by using a red color filter (right side in Figure 2(a)) located below the UV lamp or an irradiation of incandescent light onto the water. There is no color change detected and no OH^•^ generation observed inside DI water for these cases.

Figure 2(b) shows the generation of H_2_O_2_ species inside the DI water either by irradiation of UV or Ar plasma jet bombardment onto the water surface. We have put the chemical indicator of 0.01 M ammonium metavanadate (NH_4_VO_3_) into the DI water to test visually whether H_2_O_2_ has been produced in the DI and PBS. If the H_2_O_2_ has been produced in the solutions, then their colors will be changed to orange for visual confirmation. When either the Ar plasma jet or the UV has been bombarded onto the DI water surface with inclusion of 0.01 M ammonium metavanadate, we have observed that H_2_O_2_ has been generated inside the DI water. Furthermore, we could also observe H_2_O_2_ generation inside the DI water even in the case with quartz filter when the Ar plasma has been bombarded for 10 min onto the surface. The quartz filter whose thickness is 0.1 mm has been placed just 1 mm below the water surface for screening the plasma electrons, ions, and neutral particles in the plasma. In the case with quartz filter, only the plasma-initiated UVs emitted from the excited species of ROS on the surface regions could pass the filter for propagation into the DI water in this experiment. It is noted from these observations that the H_2_O_2_ and OH^•^ species could be generated simultaneously inside the DI water and PBS by plasma-initiated UVs emitted from the excited species of ROS on the surface regions.

### OH^•^ density inside the biosolutions induced by plasma-initiated UV photolysis during Ar plasma jet bombardment

Figure 2(c) shows that the OH^•^ density versus the external H_2_O_2_ concentrations 0.1%, 0.4% and 0.8% in DI water at 4 mm depth position below the DI surface when the Ar plasma jet has been bombarded onto DI surface. The driving frequency and electrical discharge power are 35 kHz and 4.9 W in this experiment. Here the OH density inside the water, produced by plasma-initiated UV photolysis, has been observed to have correlation with H_2_O_2_ concentrations. It is noted that the density of OH^•^ in DI has been increased from 1.3 × 10^16^ cm^−3^ to 2.3 × 10^16^ cm^−3^ as the H_2_O_2_ concentration in DI increases from 0% to 0.8%. Figure 2(d) also shows the density of OH^•^ versus the depth positions of the DI water and PBS solutions, generated by Ar plasma jet operated in Ar glove box. For the gas flow rate of around 250 sccm, the density of OH^•^ species in (DI, PBS) reaches the maximum value of (4.2, 1.9) × 10^16^ cm^−3^, (1.3, 0.5) × 10^16^ cm^−3^, and (0.8, 0.1) × 10^16^ cm^−3^ at depth positions of 2 mm, 4 mm, and 6 mm, respectively, below the surface. It is noted here that the densities of OH^•^ species in DI at depth regions of 2 mm, 4 mm, and 6 mm below the surface are higher by about 2 times than those in PBS, respectively. These measurement values are within ±5% error ranges.

### Influence of OH^•^ densities on lung cancer cells (H460) inside the biosolutions

We have investigated the influence of OH^•^ densities on interactions with the adhered lung cancer cells (H460) inside the PBS when the Ar plasma jet has been irradiated on PBS. The depth of PBS solution has been adjusted to be 2 mm, 4 mm, and 6 mm, and we kept the distance between the plasma plume and the solution surface be 0 mm, i.e., they are contacting to each other. After plasma exposure, cell has been divided into 96-well tissue culture test plate (30096, SPL; 1 × 10^3^cells per well) for cell death analysis^25^.

**Figure 3:**
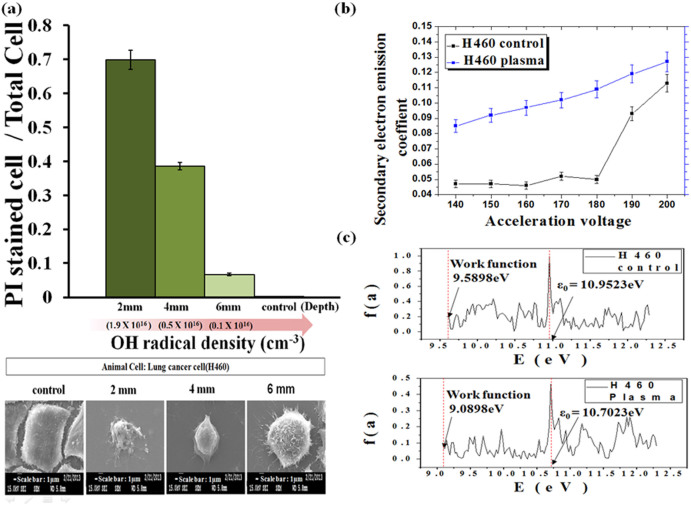
(a) Cell death area's ratio, which is calculated by ratio of cell death area of PI (Propidium Iodide: dead) stained region to NTP plasma exposed area whose diameter is 1 cm, of lung cancer H460 cells and SEM images of lung cancer H460 cells for the control and Ar plasma treatment by 60 s, adhered at 2 mm, 4 mm, and 6 mm depth positions of PBS, respectively; (b) Ion-induced secondary electron emission coefficient (γ) for lung cancer H 460 cell surfaces for the controls and plasma treated cells by 60 s, respectively, versus the incoming He ion energy ranged from 140 eV to 200 eV; (c) Molecular surface energy distribution of the lung cancer H460 cells for the control and Ar plasma treated.

Figure 3(a) shows cell death area's ratio, which is calculated by ratio of cell death area of PI (Propidium Iodide: dead) stained region to NTP plasma exposed area whose diameter is 1 cm, of lung cancer H460 cells versus depth position of 2 mm, 4 mm, and 6 mm below PBS surface, which is measured just after the Ar plasma jet treatment by 60 s. The optical microscope images for cell viability of lung cancer H460 cells by FDA (Fluorescein diacetate hydrolysis: live) and PI (Propidium Iodide: dead) staining assays are represented in Figure S2, just after the Ar plasma jet treatment for 10 s, 30 s, and 60 s, where H460 cells are adherent inside the PBS at 2 mm, 4 mm, and 6 mm depth positions. It is observed inside the PBS that the H460 cell death induced by Ar NTP jet bombardment onto the solution strongly depends on the depth position of adhered cells, which should be related to the active OH^•^ density generated by the plasma-initiated UV photolysis. For the 2 mm shallow depth position inside the PBS with OH density of ~1.9 × 10^16^ cm^−3^, the dead H460 cells have been increased to 70% as the plasma irradiation times are increased to 60 s as in Figure 3(a) (upper). Also Figure 3(a) shows SEM (scanning electron microscope) images (below) of lung cancer H460 cells for the control and Ar plasma treatment by 60 s, adhered at 2 mm, 4 mm, and 6 mm depth positions of PBS, respectively. The smooth, flat surfaces for control lung cancer cells adhered inside the PBS has been observed to be collapsed and crushed with shrunken surfaces for the shallow depth of 2 mm, while the other H460 cells located at 4 mm and 6 mm depth positions are less damaged by less number of OH^•^ densities.

It is observed in this experiment that these OH^•^ densities generated by plasma-initiated UV photolysis along with the H_2_O_2_ might induce the apoptosis of H460 cells inside the PBS under plasma irradiation from 10 s to 60 s on the solution. This kind of apoptosis could be induced by mitochondrial membrane potential (MMP) change as described in previous report^25^ and changes in the surface morphology of H460 in the PBS solution under the depth of 4 mm with OH^•^ density of about 0.5 × 10^16^ cm^−3^ under 60 s of plasma irradiation on the solution. However, there is no observation for changes in MMP and apoptosis of H460 cells under the depth of 6 mm, where the OH^•^ density is about 0.13 × 10^16^ cm^−3^ in this experiment. It is confirmed in this experiment that the threshold OH^•^ density for inducing apoptosis of H460 cells inside the PBS has been measured to be about 0.3 × 10^16^ cm^−3^ under plasma jet exposure time of 60 s on the biosolution.

Figure 3(b) shows the ion-induced secondary electron emission coefficient (*γ*) for lung cancer H460 cell surfaces for the controls (below) and plasma treated cells (upper) by 60 s, respectively, versus the incoming He^+^ ion energy ranged from 140 eV to 200 eV. The γ has been measured as an indicator for the oxidation of the cell membrane by using the He^+^ ion beam whose energy is less than 200 eV. After plasma treatment, the H460 cells were dehydrated by sequential treatment with 30, 50, 70, 80, 90, 100% ethanol (5 min/each). Treated cells have been placed on the glass plate (1 × 1 cm^2^) and dried in closed chamber at atmospheric conditions overnight. Then, analysis of the secondary electron emission coefficient has been performed on the cells. It has been observed the γ for the lung cancer H460 cells has been remarkably increased by more than 100% after plasma jet treatment in comparison with those for controls under ion energy ranges from 140 eV and 200 eV; i. e., they are increased to 0.12 for plasma treated H460 cell by 60 s from 0.05 for controls under these ion energies. It is furthermore well known and noted that the γ for the metal oxides is observed to be quite higher value of ~0.2 than those of ~0.05 for the metals under the He ion energy less than 200 eV. Hence we could say the H460 cancer cells have been highly oxidized by OH^•^ species along with the H_2_O_2_ since its γ is significantly increased by plasma treatment. Also the molecular surface energy distributions of the lung cancer H460 cells have been shown in Figure 3(c) for the control one without plasma treatment (upper), and Ar plasma treated one (below) by 60 s at 2 mm depth position in PBS with OH^•^ density of ~1.9 × 10^16^ cm^−3^, respectively.

## Discussion

**Figure 4:**
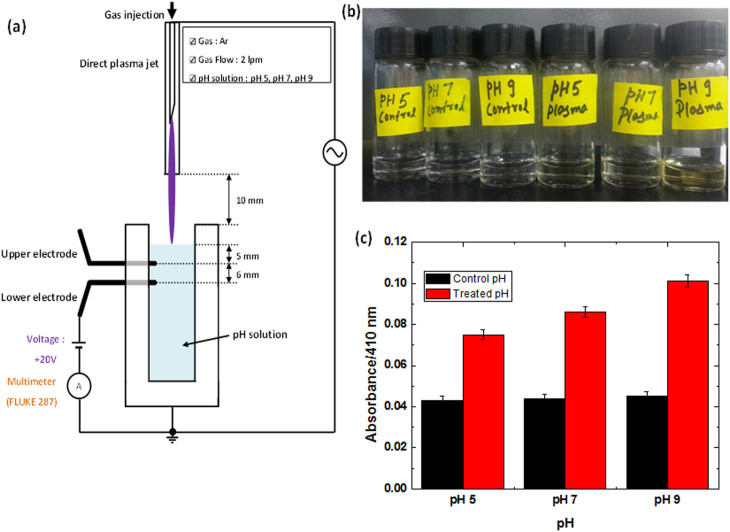
(a) Setup to determine different amount of current generated during plasma bombardment in different pH solution; (b) Visual test of H_2_O_2_ generation at different pH in the presence of titanyl ion after plasma bombardment; (c) Absorbance spectra of DI water before and after the Ar plasma treatment at different pH. To confirm the different concentration of H_2_O_2_ generation at different pH in the presence of titanyl ion.

In this work, we have tried to depict a new methodology for the generation of OH^•^ or H_2_O_2_ by plasma or UV exposure. According to our experimental results the generation of OH^•^, will be most probably through Eq. 6 in solution, that further form the H_2_O_2_. If OH^•^ is formed from OH^-^ in the solution, then there is a released electron of e^–^ into solution. Hence, to confirm the presence of e^–^, we designed a new set up, as shown in Figure 4a, where we provided 20 V in water solution at different pH (5, 7 and 9), adjusted with HCl or NaOH. This experiment reveals that high concentration of OH^–^ at pH 9 during plasma exposure resulted in more number of e^–^, i.e more current which is in correlation to Eq. 6 takes place. Table S2 shows that for both upper and lower electrodes the current is higher at pH 9 and least at pH 5; more current is obtained from the lower electrode which again shows that UV photolysis inside the water generates more e^–^. As we know that energy required for Eq. 6, is 2.4 eV, while plasma provided 4 eV energy (mention above), therefore our prediction is quite possible for generation of OH^•^.

Further, we tested the H_2_O_2_ using the titanyl ion, to confirm that presence of more concentration of OH^-^ can generate more H_2_O_2_ in the presence of plasma, through Eq. 3 or 5. Figure 4b depicts the dark yellow colour at pH 9 as compared to other pH after the plasma treatment. Similarly, Figure 4c reveals more absorbance at pH 9 due to the Ti-complex with H_2_O_2_, thus the presence of more OH^-^ (at pH 9) resulted in more generation of OH^•^ that consequently outcome in more H_2_O_2_.
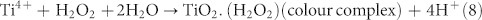


This proves our assumption that OH^-^ → OH^•^ + e^–^ is more supported for the generation of OH^•^ as compared to Eq 2.

The disappearance of OH^•^ is based on the recombination coefficient *α_OH_* which must be determined from the chemical reactions
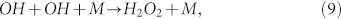
whose rate coefficient is 2.2 × 10^−11^ cm^3^/mole/s^26^,^27^ at the one atmosphere. Here, *M* represents a neutral species for this triple reaction with density of 2.5 × 10^19^/cm^3^. The other reaction is Eq. 3, whose rate coefficient is 2 × 10^−12^ cm^3^/mole/s^26^,^27^. Thus, *α_OH_* is given by *α_OH_* = 2.3×10^−11^ cm^3^/mole/s.

For the time being, we assume that the diffusion loss of OH^•^ is small and the OH^•^ have been generated during the electrical discharge, whose duration is controlled by the pulse width of the applied power module. Then, the reaction rate of OH^•^ density, *n*(r,t), can be described by
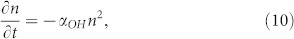


With the initial condition of *n*(*r,t*) = *n_0_*(*r*) at *t = 0*. The solution of Eq. 10 is given by
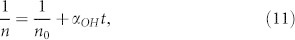
which is equivalently expressed as
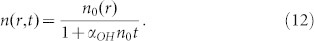


The OH^•^ density in Eq. 12 deceases inversely proportional to time *t*. Substituting *α_OH_* = 2.3 × 10^−11^ cm^3^/mole/s into Eq. 12 gives (*n/n_0_*) = 1/(1 + 2.3 × 10^−11^ *n_0_t*). Obviously, the decay time of the OH^•^ depends strongly on the initial OH^•^ density *n_0_*(*r*) at the observation position of *r*. Defining *τ* = 2/*α_OH_n_0_*, the lifetime *τ* is inversely proportional to the initial hydroxyl density *n_0_*(*r*) at the observation location of *r*. As shown in Table 1, we compared the OH^•^ life time using experimental and theoretical values. The trend of observation for lifetime is similar to the theoretical prediction, but the value of the theoretical result is somewhat different from the experimental observation, which must be explained in future studies.

The molecular surface energy distribution of H460 cell has been investigated by γ-focused ion beam (γ-FIB) device^28^ using inverse fast Fourier transform (FFT) of the energy distribution profile. Figure 3(c) explicit that the mean surface energy and work function for the plasma treated lung cancer cell decreased by 250 meV and 500 meV toward the vacuum surface level. This surface energy shift in the H460 cell induced by OH^•^ species inside the PBS could yield sufficiently high electric field E = ΔV/d = 35 MV/m between the outer and inner cell membrane, where d = 7 nm is cell membrane's thickness^29^, resulting in cell death by apoptosis due to either cell membrane distortion or mitochondria dysfunction^29^,^30^,^31^,^32^ under plasma bombardment on the solution for 60 s. The electric stress exerted on the cell surface induced by OH^•^ interactions in the PBS is ~2 kPa, which is given by (1/2)Kε_o_E^2^, where K is assumed to be 5.8 for the dielectric constant of cell bombardment^25^ of H460. This value of electric stress ~2 kPa is comparable to the mechanical stress of ~1 kPa for H460 cells^31^. Threshold OH^•^ density for apoptosis of H460 cells was found to be about 0.3 × 10^16^ cm^−3^ under PBS solution with a plasma exposure for 60 s by using a 4.9 W, 35 kHz driving frequency and 1 day incubation. Therefore, we propose the critical criterion parameter (nT) for the apoptosis of the H460 cells, in which n is OH radical density and T is effective OH^•^ exposure time, in PBS, which could be ultimately given ~2.9 × 10^16^ cm^−3^ sec.

In summary, we report that plasma-initiated UV photolysis whose energy is ranged from ~4 eV to 6 eV propagating into the solution for production of various ROS. Additionally, we have purposed the formation of OH^•^ through Eq. 6 rather than Eq. 2, by measuring the current in different pH solution in the presence of plasma exposure. Further, we also propose the critical criterion, for the apoptosis of the H460 cancer cells in PBS.

## Methods

### Ar Nonthermal plasma jet

Figure S3(a) shows the schematic needle-typed NTP jet operating at the atmospheric pressure. This plasma jet consists of needle-typed powered electrode whose diameter is 1 mm, which is located by 1 mm upward from the end of the cylindrical glass tube, whose diameter is 5 mm in diameter. Grounded electrode is located by 12 mm away from the end of inner powered electrode at the rear bottom surface of petri-dish. The biosolution such as DI water, PBS, and DMEM has been filled inside the petri dish. The distance between the powered electrode and water surface is set to be 3 mm. For investigation of ROS generation mechanism inside the solutions, the NTP jet has been bombarded onto the solution. We have used an argon gas flow in this experiment for the generation of NTP jet. The two electrodes in a NTP jet have been connected to a square-pulse power supply. It is noted that the voltage waveform applied to the non-thermal plasma jet is square pulse, and the discharge current has very short waveforms in comparison with those of voltage. It is shown that the voltage signal, *V*, has a root-mean-square voltage of about 1 kV and peak current, *I*, of 34 mA, with current duration of about 2.1 us, where the electrical power is given by about 4.9 W, which is obtained from 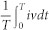, where T is period of squared pulse with the repetition rate of about 35 kHz.

The electron temperature and ion density for this non-thermal plasma jet have been measured to be about 0.8 ~ 1.0 eV and ~2 × 10^13^ cm^−3^ in this experiment, respectively, by atmospheric pressure collisional radiative model and ion collector current^7^. Additionally, the ROS measurement by optical emission and ultraviolet absorption spectroscopy above and below the biosolutions, γ-FIB system and the secondary electron emission coefficient, and Lung cancer cell (H460) culture and related experiments are provided in the supporting file. The structures of OH radical and water have been optimized based on molecular mechanics and semi-empirical calculations using the HyperChem 7 molecular visualization and simulation program^22^,^23^.

### ROS measurement by optical emission and ultraviolet absorption spectroscopy above and below the biosolutions

The optical emission and ultraviolet absorption spectroscopy used for ROS measurement above and below the biosolutions, as shown in Figure S3b. This system consists of reference deuterium UV lamp in this experiment, whose power is 30 W with spectral wavelength between 160 nm and 800 nm, plano-convex lens whose transmission wavelength ranges are from ultraviolet to infrared. The deuterium lamp has a continuous spectrum from 200 nm to 500 nm and the spectral range for CCD is from 200 to 1100 nm in this experiment. To get an absorption spectrum caused by the OH^•^ species inside the solutions, two plano-convex lenses have been used for providing parallel UV lights produced from the deuterium lamp and then making a crossing beam with focused diameter of 200 um when transmission through a middle position of the biosolution, whose passing space is 10 mm. It is noted that the UV beam diameter becomes to be 500 um for its collection to the double slit and collimator lens connected to the optical fiber and CCD spectrometer for the measurement of absorption profiles occurred especially at 309 nm of the OH^•^ species, as shown in the Figure S3(b). These absorption optical signals could be observed by monochromator or charge-coupled device (CCD) spectrometer connected by the optical fiber with double slit and a collimator lens. The double slit width is set to be 100 um and their separation is 1 mm to prevent the optical stray signal caused by the light scattering in this experiment. The optical lens system for deuterium lamp has been fixed during the measurement. However, for the measurement of OH^•^ species at the different position inside the biosolution, the optical detection system for double slit, collimator lens, and optical fiber could be moved downward together along the depth direction with spatial resolution of about 1 mm since the output UV has beam diameter of around 500 um.

The argon glove box has been used in this experiment as shown in the dotted box region of Figure S3(b) to eliminate the optical signals from N_2_ molecules close to 309 nm, which is the emission and absorption line of the OH^•^ species, caused by the Ar NTP jet. Also, we have measured the reactive oxygen species, especially, for the OH^•^ in this paper, qualitatively, by the optical emission spectroscopy, as well as their absolute densities, quantitatively, inside the solution by the ultraviolet (UV) absorption spectroscopy when the nonthermal plasma has been bombarded onto the solution surface. The incident deuterium light on the biosolution, in which the OH^•^ species and H_2_O_2_ are generated during the nonthermal plasma bombardment onto the solution surface, has the intensity *I_o_* and the transmitted light intensity is denoted by *I_V_* after passing through the OH^•^ existing region, *x* inside the biosolution. The UV light intensity has been absorbed by OH^•^ species, along the light passing region *x* = 10 mm inside the solutions. The density of OH^•^ species, inside the biosolution, which are generated by the nonthermal plasma bombardment onto the solution, is given by
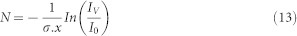


Where *N* is the density for absorbing species of either OH^•^ or other ROS, *σ* is the cross sectional area whose value is about 0.6 × 10^−16^ cm^2^ for hydroxyl OH radical species. The OH^•^ density inside the biosolution could be obtained by Eq. [13], by measurement of *I_V_*/*I_o_*, which is the ratio of the transmitted intensity *I_V_* to the incident one *I_o_* during the nonthermal plasma irradiation onto the solution.

### γ-focused ion beam (γ-FIB) system and the secondary electron emission coefficient

For observing the changes in the secondary electron emission coefficient, γ, and molecular energy band structure of the biological cells caused by atmospheric nonthermal plasma treatment, the γ-focused ion beam (γ-FIB) system^28^ has been used in this experiment. Figure S3(c) shows a schematic view of the γ-focused ion beam (FIB) consisted of the thermal electron source, the ionization region of ions, the electrostatic single Einzel lens that focused the ion beam, and the collector and copper pad for the measurement of the secondary electron emissions from the surface of the biological thin films. We have employed the He^+^ ion whose ionization energy E_i_ is 24.58 eV with low energy below 200 eV in this experiment. The He^+^ ion approaches the surface of biological film. It is noted that the electric field is polarized toward the collector from the grounded copper pad when the collector potential is negatively biased. The secondary electrons emitted from the surface by this slow ion then come back, and only the ion current (I_i_) coming to the surface is then measured. On the other hand, the positively biased collector makes the electric field toward the grounded copper pad. Due to this electric field, the secondary electrons emitted from the surface of thin biological film by the ion beam bombardment move up toward the collector, registering the current (I_t_) in the ampere meter shown in the Figure 1, in which the emitted secondary electron and ion beam currents (I_i_) are included together. The secondary electron emission γ is obtained from γ = (I_t_-I_i_)/I_i_.

### Lung cancer cell (H460) culture and related experiments

Lung epithelial cancer cell line H460 has been purchased from Korean Cell Line Bank (Korea). Cells were maintained in high glucose DMEM (SH30243.01, Hyclone) supplemented with 10% FBS (SH30979.03, Hyclone), 1% of Penicillin/Streptomycin (15140, Gibco). For plasma exposure, 1 × 10^5^cells were seeded at 24 well-plate (SPL, Korea) 24 hrs before and 500, 1000, and 1500 μl of PBS were added right before plasma exposure for keeping height of solution 2, 4, and 6 mm between cells and gas plasma, respectively. The distance between the powered electrode and the surface of media was kept approximately 2 mm. The plasma exposure time has been varied from 10 s to 60 s for viability test, and 60 s exposed samples have been used for further experiment such as γ- FIB and SEM analysis.

After plasma exposure, cells have been stained by FDA (Fluorescein diacetate; F7378, Sigma) and PI (Propidium iodide; P4170, Sigma) to distinguish viable and dead cells. Fluorescent images have been directly taken from well plate by using fluorescence inverted microscope (Ti-U, Nikon). For SEM analysis, plasma treated cells have been fixed with Karnovsky's fixative (18505, TedPella and 18420, TedPella) and osmium tetroxide (18450, TedPella) and dehydrated by ethanol series. Finally, cells have been dehydrated in HMDS (hexamethyldisilazane; H00326, Lancaster) solution to reduce morphological deformation, and observed by using SEM machine (JEOL 7001F). For γ-FIB analysis, cells have been dehydrated without fixation to reduce further membrane oxidation. After series of ethanol, secondary electron emission has been measured by γ-FIB device.

## Author Contributions

E.H.C., A.F. and K.N. conceived and designed the experiments. P.A. performed the semiempirical research and current generated experiment in different pH solution. Y.H.K. and Y.J.H. analyzed the Optical emission spectrum. D.H.P. provided assistance with designing cell cycle experiments. J.H.P. provided assistance with current generated experiment. H.S.U. provide the interpretation and analysis of the data E.H.C. and P.A. conceived and wrote manuscript.

## Supplementary Material

Supplementary Information Supplementary Information

## Figures and Tables

**Table 1:** Comparison between theory and experimental values of OH^•^ lifetime

OH density (×10^16^/cm^3^)	Experimental (*μs*)	Theory (*μs*)
4.2	3.15	2.06
1.8	3.61	4.82
0.8	3.92	10.8
