# Supplementary material for: Generation mechanism of hydroxyl radical species and its lifetime prediction during the plasma-initiated ultraviolet (UV) photolysis
Source: Sci Rep. 2015 Mar 20;5:9332. doi: 10.1038/srep09332 (PMC4367430; doi:10.1038/srep09332)
Supplement: Supplementary Information [file srep09332-s1.pdf]

Supporting file

Generation mechanism of hydroxyl radical species and its lifetime prediction during the plasma-initiated ultraviolet (UV) photolysis

Pankaj Attri[a], Yong Hee Kim[a], Dae Hoon Park[a], Ji Hoon Park[a], Young J. Hong[a], Han Sup Uhm[a], Kyung Nam Kim[b] Alexander Fridman[c] and Eun Ha Choi*[a]

[a]*Plasma Bioscience Research Center, Kwangwoon University, Seoul, Korea.*

[b]*Yonsei University, Seoul, Korea.*

[c]*Drexel Plasma Institute, Drexel University, PA, USA.*

Supporting Figure Captions

Figure S1. Hydrogen bonding calculation between OH radicals and H_2O using semiempirical calculations with the help of Hyperchem 7. We have chosen the (a) 2 OH radical and 40 water molecules for the first calculation. This is try to resemble the inside depth of water ~ 6 mm, where less OH radical and more water molecules (assumption); (b)We choose the 4 OH radical and 40 water molecules to resemble the condition at ~ 4 mm (assumption).

Figure S2. Optical microscope images for cell viability of lung cancer H460 cells by FDA (Fluorescein diacetatehydrolysis : live) and PI (Propidium Iodide : dead) staining assays just after the Ar plasma jet treatment for 10 s, 30 s, and 60 s, where H460 cells are adherent inside the PBS at 2 mm, 4 mm, and 6 mm depth positions.

Figure S3 (a) Schematic needle-typed nonthermal plasma jet operating at the atmospheric pressure; (b) Optical emission and ultraviolet absorption spectroscopy used for ROS measurement above and below the solutions. The argon glove box has been used as shown in the dotted box region to eliminate the optical signals from N_2 molecules in air caused by the nonthermal Ar plasma jet, close to 309 nm, which is the emission and absorption line of the hydroxyl OH species; (c) Schematic view of the γ-focused ion beam (γ-FIB) consisting of the thermal electron source, the ionization region of ions, the electrostatic single Einzel lens that focused the ion beam, and the collector and copper pad for the measurement of the secondary

electron emissions from the surface of the biological thin films.

Table S1. Calculated binding energies (E), heats of formations (ΔH_f), and estimated hydrogen bond energies ($\Delta\Delta H_f$) (kcal /mol)

Solvent	E/ (kcal /mol)	ΔH_f/ (kcal /mol)	$\Delta\Delta H_f$/ (kcal /mol)
2 OH radical	-197.378	8.511	
40 water	-8837.639	-2123.356	
2 OH radical and 40 water	-9196.080	-2258.470	126.603
4 OH radical	-393.509	18.706	
4 OH radical and 40 water	-9176.866	-2179.702	37.64

Table S2: Current measurements in different pH solution during plasma exposure.

**pH**	**Voltage**	**Electrode**	**Current Average (mA)**	**Gap (mA)**
pH 5	20V	Upper	1.606	0.0755
		Lower	1.681	
pH 7	20V	Upper	1.547	0.0990
		Lower	1.646	
pH 9	20V	Upper	1.326	0.1785
		Lower	1.504	

(a)

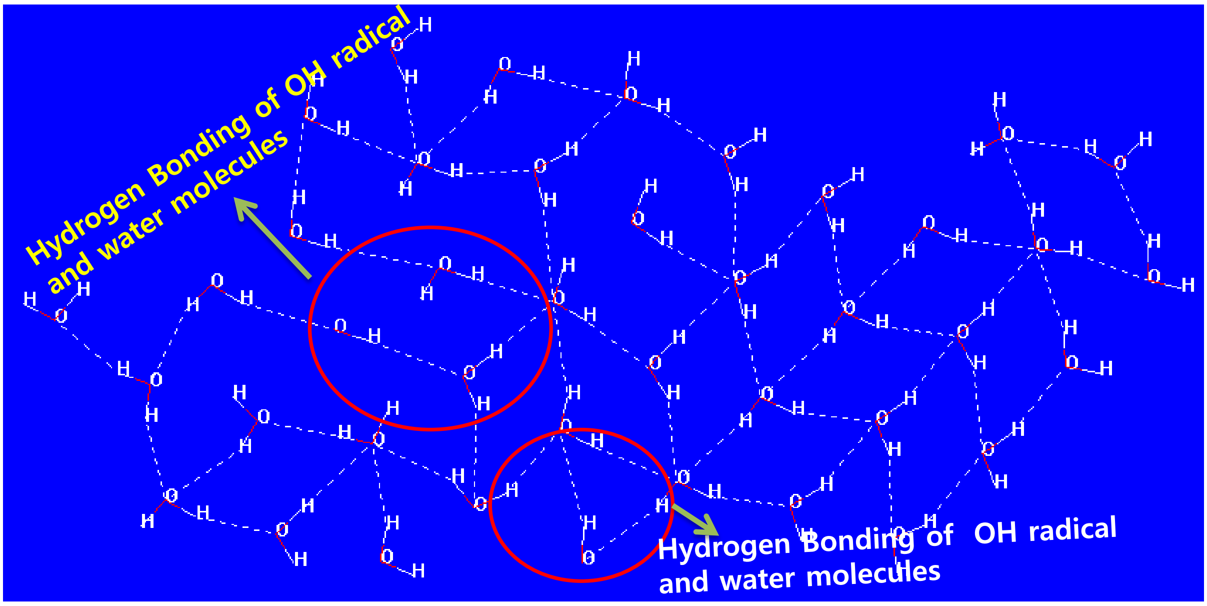
Hydrogen Bonding of OH radical and water molecules
Hydrogen Bonding of OH radical and water molecules

(b)

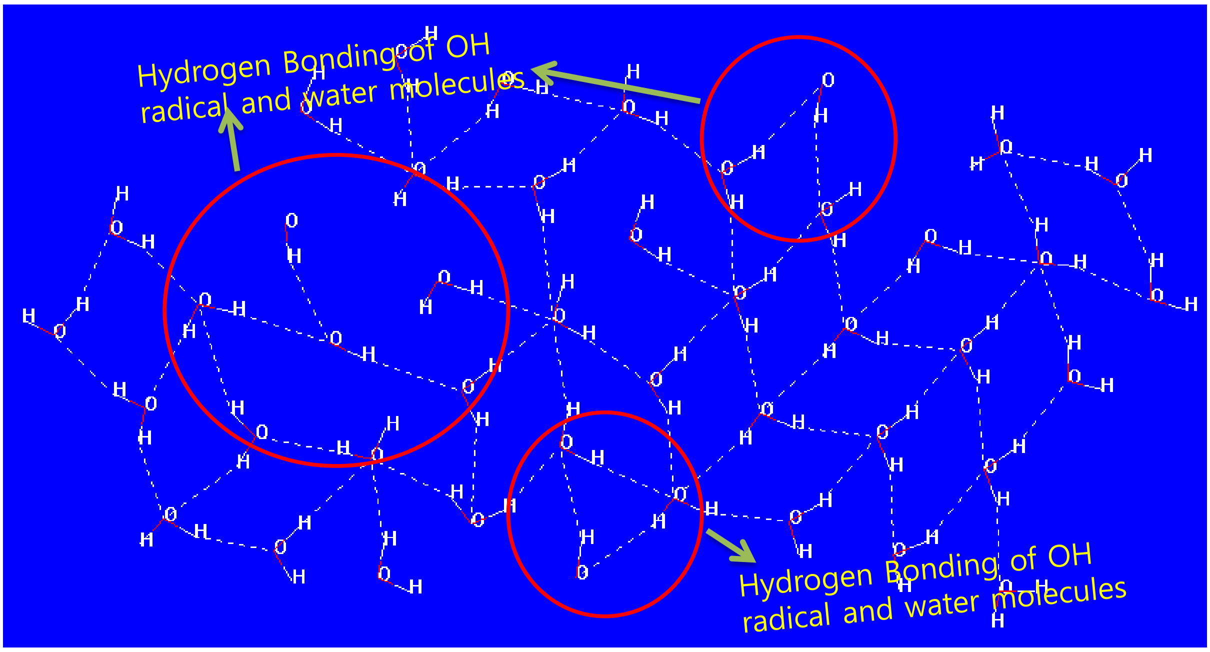
Hydrogen Bonding of OH radical and water molecules
Hydrogen Bonding of OH radical and water molecules

Figure S1.

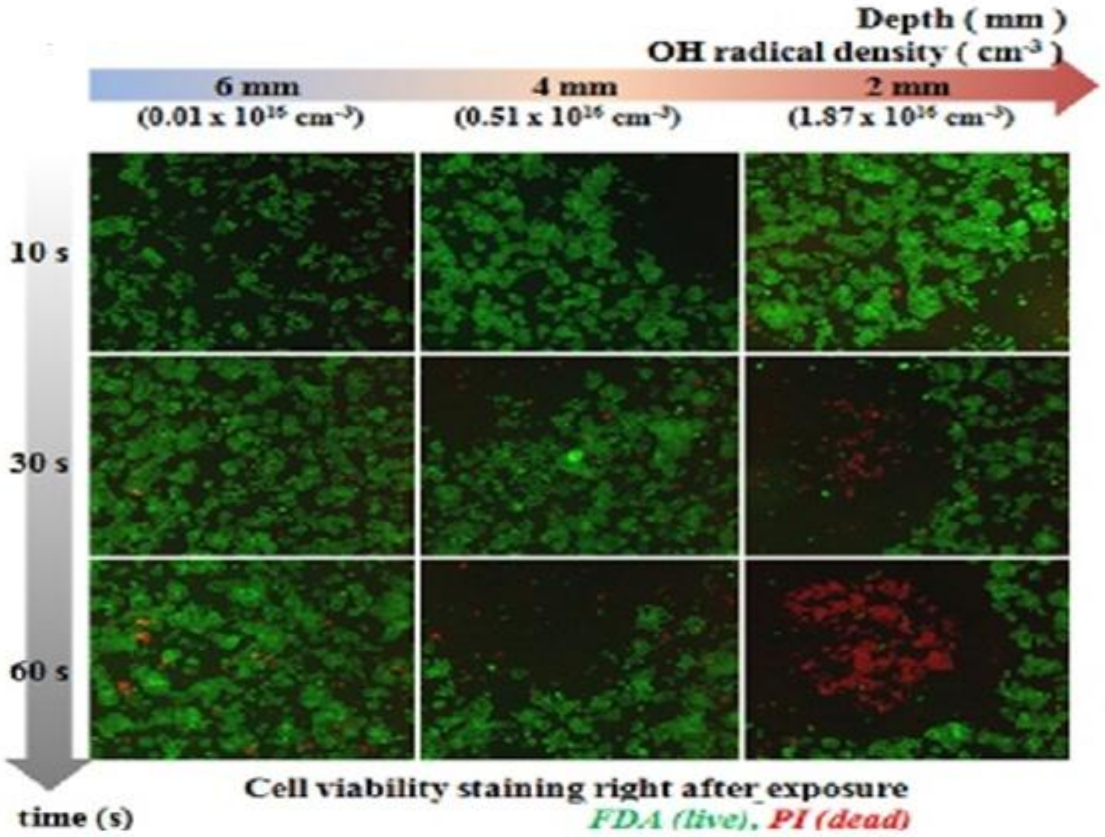
Depth (mm)
OH radical density (cm⁻³)
6 mm
(0.01 x 10¹⁶ cm⁻³)
4 mm
(0.51 x 10¹⁶ cm⁻³)
2 mm
(1.87 x 10¹⁶ cm⁻³)
10 s
30 s
60 s
time (s)
Cell viability staining right after exposure
FDA (live), PI (dead)

Figure S2

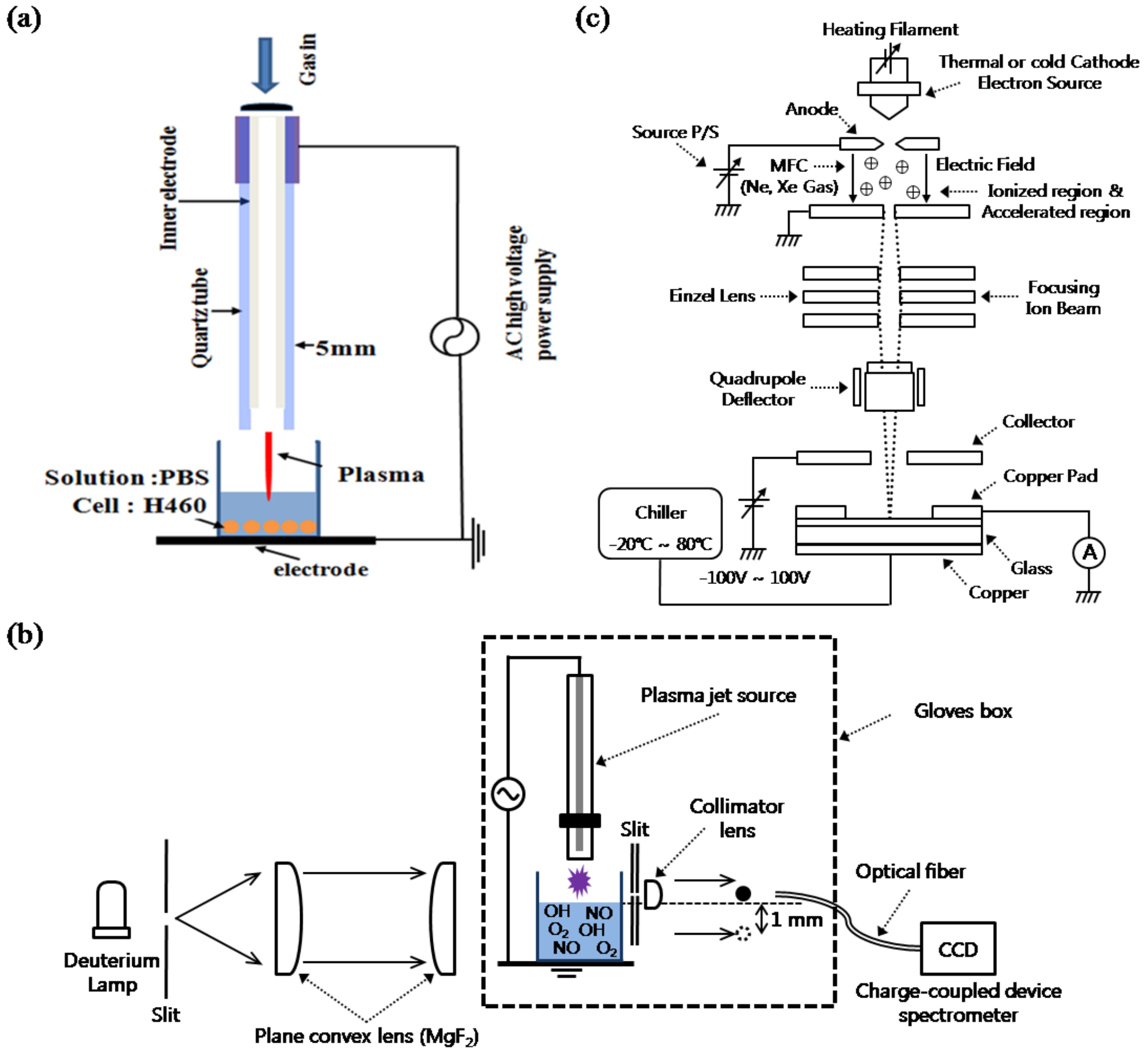

(a)
Gas in
Inner electrode
Quartz tube
5mm
AC high voltage power supply
Solution :PBS
Cell : H460
Plasma
electrode
(c)
Heating Filament
Thermal or cold Cathode Electron Source
Anode
Source P/S
MFC
(Ne, Xe Gas)
Electric Field
Ionized region &
Accelerated region
Einzel Lens
Focusing Ion Beam
Quadrupole Deflector
Collector
Copper Pad
Chiller
-20℃ ~ 80℃
-100V ~ 100V
Glass
Copper
A
(b)
Plasma jet source
Gloves box
Collimator lens
Slit
OH NO O2 OH NO O2
1 mm
Optical fiber
CCD
Charge-coupled device spectrometer
Deuterium Lamp
Slit
Plane convex lens (MgF2)

Figure S3
